# Supplementary material for: Predicting visual function by interpreting a neuronal wiring diagram
Source: Nature. 2024 Oct 2;634(8032):113–23. doi: 10.1038/s41586-024-07953-5 (PMC11446822; doi:10.1038/s41586-024-07953-5)
Supplement: Supplementary file 6 — CRF and ERF predictions for individual TmY4 and TmY9 cells. Analogous to Supplementary Data 3, but for TmY target types. Shown are the top four monosynaptic pathways, the strongest pathway passing through each of the top ten intermediary types (ranking from Extended Data Fig. 7), and the trisynaptic pathway Tm1–TmY–Dm3–TmY (see the section entitled Prediction of spatial normalization). [file 41586_2024_7953_MOESM6_ESM.zip › DataS4/TmY9q/720575940635299167.pdf]

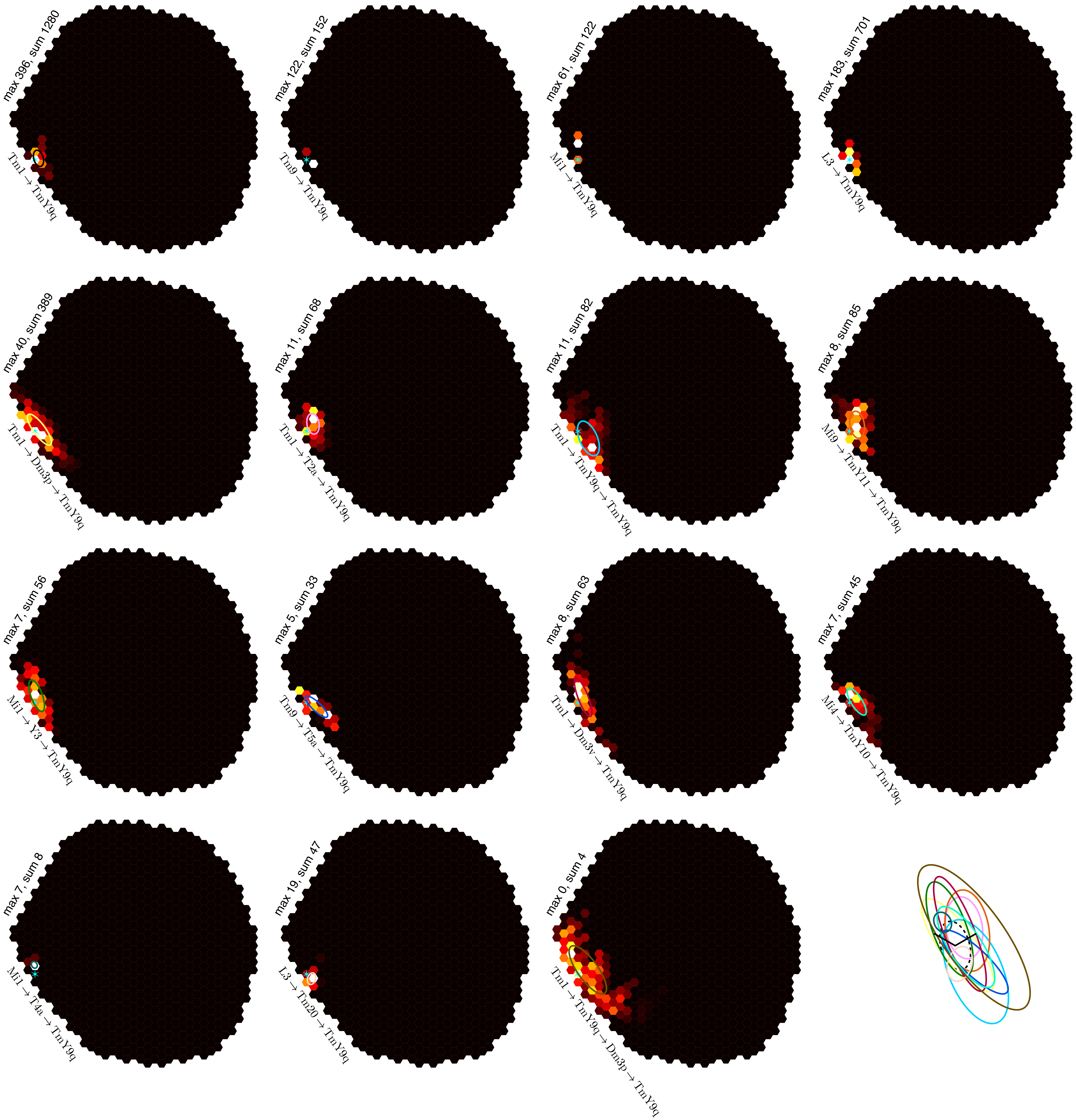
max 396, sum 1280
Tm1 → TmY9q
max 122, sum 152
Tm9 → TmY9q
max 61, sum 122
Mi1 → TmY9q
max 183, sum 701
L3 → TmY9q
max 40, sum 389
Tm1 → Dm3p → TmY9q
max 11, sum 68
Tm1 → T2a → TmY9q
max 11, sum 82
Tm1 → TmY9q → TmY9q
max 8, sum 85
Mi9 → TmY11 → TmY9q
max 7, sum 56
Mi1 → Y3 → TmY9q
max 5, sum 33
Tm9 → T5a → TmY9q
max 8, sum 63
Tm1 → Dm3v → TmY9q
max 7, sum 45
Mi4 → TmY10 → TmY9q
max 7, sum 8
Mi1 → T4a → TmY9q
max 19, sum 47
L3 → Tm20 → TmY9q
max 0, sum 4
Tm1 → TmY9q → Dm3p → TmY9q
